# Dietary fatty acids intake and endometrial cancer risk: a dose-response meta-analysis of epidemiological studies

**DOI:** 10.18632/oncotarget.5555

**Published:** 2015-10-07

**Authors:** Qi-Jun Wu, Ting-Ting Gong, Ya-Zhu Wang

**Affiliations:** ^1^ Department of Clinical Epidemiology, Shengjing Hospital of China Medical University, Shenyang, China; ^2^ Department of Obstetrics and Gynecology, Shengjing Hospital of China Medical University, Shenyang, China; ^3^ Department of Hematology, The First Affiliated Hospital of China Medical University, Shenyang, China

**Keywords:** endometrial cancer, epidemiology, fatty acids, meta-analysis

## Abstract

Epidemiological studies have provided controversial evidence of the association between dietary fatty acids intake and endometrial cancer risk. The continuous update project of World Cancer Research Fund failed to focus on this issue. To address this inconsistency, we conducted this dose-response meta-analysis based on epidemiological studies published up to the end of June 2015 identified from PubMed, EMBASE and Web of Science. Two authors independently performed the eligibility evaluation and data extraction. Random-effects models were used to estimate summary relative risks (RRs) and 95% confidence intervals (CIs). Fourteen epidemiological studies (4 cohort and 10 case-control studies) were included in this dose-response meta-analysis. The summary RR for an intake increment of 10g/day was 1.02 (95% CI = 0.97–1.08; *I*^2^ = 66.0%) for saturated fatty acids, 0.98 (95% CI = 0.96–1.001; *I*^2^ = 0%) for monounsaturated fatty acids, and 1.00 (95% CI = 0.95–1.06; *I*^2^ = 0%) for polyunsaturated fatty acids intake. Non-significant results were observed in the majority of subgroup analyses stratified by study characteristics and adjustment for potential confounders in analyses of aforementioned associations. In conclusion, results from this dose-response meta-analysis provided limited evidence that dietary saturated, monounsaturated, and polyunsaturated fatty acids consumption was associated with endometrial cancer risk. Further studies, especial prospective designed or pooled studies are warranted to confirm our findings.

## INTRODUCTION

About 320,000 new cases of endometrial cancer (EC) were diagnosed and nearly 76,000 deaths from this disease occurred worldwide in 2012 [1]. By comparison with Africa and South Asia, the incidence rates of this disease were higher in North America and Europe [1]. Nonetheless, this discrepancy could not be totally attributed to these well established risk factors including obesity, reproductive factors (e.g., parity, age at menarche), and use of exogenous hormones (e.g., estrogen hormonal replacement therapy, oral contraceptives) [2]. During the past decade, experimental and epidemiological studies have suggested that dietary factors may contribute to the etiology of EC because diet might be an important difference of lifestyle of these countries [2].

*In vitro* and *in vivo* studies have indicated that dietary fat and fatty acids (FA) intake have been proposed to influence EC risk by modulating the production, metabolism, and excretion of endogenous hormones [3–7]. However, the continuous update project of World Cancer Research Fund and American Institute for Cancer Research (WCRF/AICR) including studies up to December 2012 only investigated the association between total dietary fat intake and EC risk which indicated limited evidence [8]. The relationships between dietary different FA (saturated, monounsaturated, and polyunsaturated FA) intake and risk of EC have remained inconsistent and elusive which were not summarized in this updated report [7–20]. In 2007, a meta-analysis including 7 studies (one cohort and 6 case-control studies) showed a relative risk (RR) of 1.49 (95% confidence interval (CI) = 1.11–2.01, *I*^2^ = 52.7%, *P* for heterogeneity = 0.06) for the highest compared with the lowest intakes of dietary saturated FA [21]. However, the results were hard to interpret because the definitions of the categories differed among each study [21]. During the recent five years, the findings from one of the largest population-based cohort studies, the European Prospective Investigation into Cancer and Nutrition (EPIC) found non-significant result of saturated and polyunsaturated FA but suggested significant result of monounsaturated FA intake with EC risk [9]. In contrast, the Nurses’ Health Studies (NHS/NHSII) updated their evidence but found non-significant association between monounsaturated FA intake and EC risk [9]. Additionally, to our knowledge, a comprehensive and quantitative assessment of the relationship between dietary FA intake and EC risk has not been reported. Therefore, we carried out this dose-response meta-analysis of epidemiological studies to assess the aforementioned associations.

## RESULTS

### Search results, study characteristics, and quality assessment

**Figure 1:**
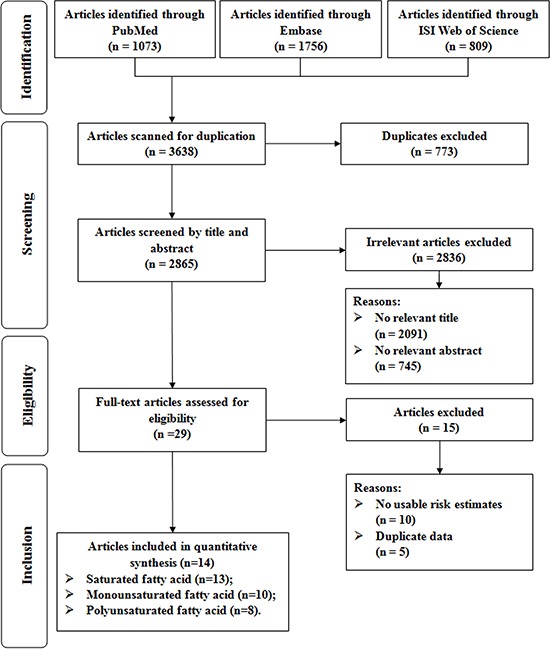
Flow-chart of study selection

Figure 1 presented the detailed procedures of the article search and screening. Briefly, the search strategy retrieved 3638 articles: 1073 from PubMed, 1756 from EMBASE, and 809 from Web of Science. Of these, 3609 articles were excluded after the first screening based on abstracts or titles, leaving 29 articles for full-text review. Among them, fifteen articles were further excluded due to i) no usable risk estimates or 95%CIs were reported; and ii) study population duplication. Overall, a total of 13 articles (14 studies) were included in the present meta-analysis [7, 9–20].

Table 1 demonstrated the characteristics of the 14 selected studies. These studies were published between 1993 and 2015 and involved a total of 7741 EC cases and 583,892 non-cases. There were 4 cohort and 10 case-control studies. Of the 4 cohort studies, three were conducted in North America and one in Europe. Of the 10 case-control studies, eight were conducted in North America and two in Europe. Age adjusted risk estimates could be determined for all studies. Risk measures were also adjusted for body mass index (13 studies), total energy intake (13 studies), parity (12 studies), hormone replacement therapy (12 studies), oral contraceptive use (11 studies), cigarette smoking (11 studies), and menopausal status (9 studies).

**Table 1:** Characteristics of studies included in the dose-response meta-analysis

First author (ref), year, Country	No. of cases/cohort, age, follow-up	Energy-adjusted model (unit)	Exposure categories (Dietary assessment)	Risk estimates (95% CI)	Matched/adjusted factors
**Prospective study**					
Merritt et al [9], 2015, Europe	1303/301,107 (25–70y), 11y	Residual (g/day)	**Quartile 4 *vs*. Quartile 1** Saturated fat Polyunsaturated fat Monounsaturated fat (Validated FFQ)	**Hazard ratio** 0.95 (0.81–1.12) 0.87 (0.73–1.03) 0.80 (0.65–0.97)	BMI, total energy intake, smoking status, age at menarche, OC use, parity, and a combined variable for menopausal status and postmenopausal hormone use and were stratified by age and study center
Merritt et al [9], 2015, USA	1531/155,406 (30–55y), 25y	Residual (g/day)	**Quartile 4 *vs*. Quartile 1** Monounsaturated fat (Validated FFQ)	**Hazard ratio** 0.95 (0.81–1.10)	BMI, total energy intake, smoking status, age at menarche, OC use, parity, and menopausal status and postmenopausal hormone use and were stratified by age, cohort, and the 2-year questionnaire cycle
Cui et al [7], 2011, USA	669/68,070 (30–55y), 21y	N/A (g/day)	**Quintile 5 *vs*. Quintile 1** Saturated fat Polyunsaturated fat (Validated FFQ)	**Relative Risk** 0.90 (0.70–1.16) 1.05 (0.82–1.34)	Age, follow-up period, total energy, smoking, OC use, postmenopausal hormone use, age at menopause, parity, age at menarche, hypertension, diabetes, BMI
Jain et al [16], 2000, Canada	221/56,837 (40–59y), 9y	Residual (g/day)	**Quartile 4 *vs*. Quartile 1** Saturated fat (Validated FFQ)	**Relative Risk** 0.70 (0.47–1.04)	Total energy, age, BMI, ever smoked, used OC, used HRT, university education, livebirths, age at menarche
**Case-control study**					
Biel et al [10], 2011, Canada, PC-CS	506/981 (mean, 58.7/58.3y)	Residual (g/day)	**Quartile 4 *vs*. Quartile 1** Saturated fat Monounsaturated fat Polyunsaturated fat (Validated FFQ)	**Odds Ratio** 1.06 (0.76–1.49) 1.07 (0.77–1.48) 1.11 (0.81–1.54)	Age, total energy intake, age at menarche, BMI, parity, educational level, hypertension history, OC use, HRT use combined with menopausal status, and alcohol consumption
Chandran et al [11], 2010, USA, PC-CS	424/398 (mean, 61.6/64.3y)	N/A (g/day)	**Quartile 4 *vs*. Quartile 1** Saturated fat(FFQ)	**Odds Ratio** 0.82 (0.43–1.58)	Age, education, race, age at menarche, menopausal status and age at menopause for postmenopausalwomen, parity, OC use, HRT use, BMI, total calories intake, PA, smoking status, and alcohol
Yeh et al [12], 2009, USA, HC-CS	541/541 (mean, 63.3/63.2y)	N/A (g/day)	**Quartile 4 *vs*. Quartile 1** Saturated fat Monounsaturated fat Polyunsaturated fat (FFQ)	**Odds Ratio** 1.51 (0.84–2.68) 1.26 (0.71–2.23) 0.85 (0.51–1.41)	Age, BMI, exogenous estrogen use, smoking, total menstrual months, total energy, total protein and carbohydrates intake
Lucenteforte et al [13], 2008, Italy, HC-CS	454/908 (median, 60/61y)	Residual (g/day)	**Quintile 5 *vs*. Quintile 1** Saturated fat Monounsaturated fat Polyunsaturated fat (Validated FFQ)	**Odds Ratio** 1.30 (0.90–2.00) 0.80 (0.60–1.20) 0.90 (0.60–1.40)	Age, study centre, year of interview, education, PA, BMI, history of diabetes, age at menarche, age at menopause, parity, OC use, HRT use, total energy intake, according to the residual models
Salazar-Martinez et al [14], 2005, Mexico, HC-CS	85/629 (mean, 51.7/57.1y)	Residual (g/day)	**Tertile 3 *vs*. Tertile 1** Saturated fat Monounsaturated fat Polyunsaturated fat (Validated FFQ)	**Odds Ratio** 0.67 (0.29–1.53) 0.98 (0.41–2.43) 2.23 (0.98–5.05)	Age, total energy intake, number of live births, BMI, PA, and diabetes
Littman et al [15], 2001, USA, PC-CS	679/944 (45–74y)	Presented(g/1000 kcal/d)	**Quintile 5 *vs*. Quintile 1** Saturated fat (FFQ)	**Odds Ratio** 1.60 (1.10–2.30)	Age, county of residence, total energy intake, unopposed estrogen use, cigarette smoking, and BMI
McCann et al [17], 2000, USA, PC-CS	232/639 (mean, 63.5/55.9y)	N/A (g/day)	**Quartile 4 *vs*. Quartile 1** Saturated fat Monounsaturated fat Polyunsaturated fat (Validated FFQ)	**Odds Ratio** 1.10 (0.50–2.40) 0.90 (0.40–1.80) 1.00 (0.50–1.80)	Age, education, BMI, diabetes, hypertension, pack-years cigarette smoking, age at menarche, parity, OC use, menopause status, postmenopausal estrogen use, and total energy intake
Jain et al [18], 2000, Canada, PC-CS	552/562 (30–79y)	Residual (g/day)	**Quartile 4 *vs*. Quartile 1** Saturated fat Monounsaturated fat (Validated FFQ)	**Odds Ratio** 1.22 (0.85–1.75) 1.08 (0.76–1.53)	Total energy, age, body weight, ever smoked, history of diabetes, used OC, used HRT, university education, live births, age at menarche
Tzonou et al [19],* 1996, Greece, HC-CS	145/298 (N/A)	N/A (g/day)	**Quartile 4 *vs*. Quartile 1** Saturated fat Monounsaturated fat Polyunsaturated fat (FFQ)	**Odds Ratio** 0.75 (0.43–1.30) 0.67 (0.39–1.16) 1.13 (0.63–2.01)	Age
Potischman et al [20], 1993, USA, PC-CS	399/296 (mean, 59.1/58y)	N/A (g/day)	**Quartile 4 *vs*. Quartile 1** Saturated fat (FFQ)	**Odds Ratio** 2.10 (1.20–3.70)	Age, BMI, current smoking, years of education, number of births, ever OC use, ever menopausal estrogen use, and total calories intake

BMI, body mass index; CI, confidence interval; HC-CS, hospital-based case-control study; PA, physical activity; PC-CS, population-based case-control study; N/A, not available; OC, oral contraceptive; FFQ, food frequency questionnaire.

* Risk estimates were calculated from published data with EpiCalc 2000 software (version 1.02; Brixton Health).

The information of study quality assessment is demonstrated in Table 2 and Table 3. Briefly, for the category of ‘follow-up long enough for outcomes to occur’, all cohort studies were assigned a score except one study [16] because the mean follow-up period was less than 10 years. For the category of ‘using an energy-adjusted model’, one study [7] failed to carry out it in their analysis (Table 2). Furthermore, for the category of ‘selection of control subjects’, four case-control studies [12–14, 19] were not assigned a score because the controls of their study were not from populations; For the category of ‘control for important factor or additional factor’, all case-control studies were assigned two scores except one study [19] because they adjusted for less than two important confounders in their multivariable analysis; For the category of ‘exposure assessment’, five case-control studies [10, 13, 14, 17, 18] were assigned a score because their FFQs were validated. Five-case-control studies were assigned a score because there was no difference of response rate between cases and controls [11, 13, 15, 17, 19]. Five case-control studies [10, 13–15, 18] were assigned a score because they presented or considered energy-adjusted model in their primary analyses (Table 3).

**Table 2:** Methodological quality of prospective studies included in the meta-analysis*

First author (reference), publication year	Representativeness of the exposed cohort	Selection of the unexposed cohort	Ascertainment of exposure	Outcome of interest not present at start of study	Control for important factor or additional factor^†^	Assessment of outcome	Follow-up long enough for outcomes to occur^‡^	Adequacy of follow-up of cohorts^§^	Using an energy-adjusted model
Merritt et al [9], 2015	☆	☆	☆	☆	☆	☆	☆	☆	☆
Merritt et al [9], 2015	☆	☆	☆	☆	☆	☆	☆	☆	☆
Cui et al [7], 2011	☆	☆	☆	☆	☆	☆	☆	☆	—
Jain et al [16], 2000	☆	☆	☆	☆	☆	☆	—	☆	☆

* A study could be awarded a maximum of one star for each item except for the item Control for important factor or additional factor. The definition/explanation of each column of the Newcastle-Ottawa Scale is available from (http://www.ohri.ca/programs/clinical_epidemiology/oxford.asp.).

† A maximum of 2 stars could be awarded for this item. Studies that controlled for total energy intake received one star, whereas studies that controlled for other important confounders such as body mass index, reproductive factors received an additional star.

‡ A cohort study with a follow-up time > 10 y was assigned one star.

§ A cohort study with a follow-up rate > 75% was assigned one star.

**Table 3:** Methodological quality of case-control studies included in the meta-analysis*

First author (reference), publication year	Adequate definition of cases	Representativeness of cases	Selection of control subjects	Definition of control subjects	Control for important factor or additional factor^†^	Exposure assessment	Same method of ascertainment for all subjects	Non response Rate^‡^	Using an energy-adjusted model
Biel et al [10], 2011	☆	☆	☆	☆	☆	☆	☆	—	☆
Chandran et al [11], 2010	☆	☆	☆	☆	☆	—	☆	☆	—
Yeh et al [12], 2009	☆	☆	—	☆	☆	—	☆	—	—
Lucenteforte et al [13], 2008	☆	☆	—	☆	☆	☆	☆	☆	☆
Salazar-Martinez et al [14], 2005	☆	☆	—	☆	☆	☆	☆	—	☆
Littman et al [15], 2001	☆	☆	☆	☆	☆	—	☆	☆	☆
McCann et al [17], 2000	☆	☆	☆	☆	☆	☆	☆	☆	—
Jain et al [18], 2000	☆	☆	☆	☆	☆	☆	☆	—	☆
Tzonou et al [19], 1996	☆	☆	—	☆	—	—	☆	☆	—
Potischman et al [20], 1993	☆	☆	☆	☆	☆	—	☆	—	—

* A study could be awarded a maximum of one star for each item except for the item Control for important factor or additional factor. The definition/explanation of each column of the Newcastle-Ottawa Scale is available from (http://www.ohri.ca/programs/clinical_epidemiology/oxford.asp.).

† A maximum of 2 stars could be awarded for this item. Studies that controlled for total energy intake received one star, whereas studies that controlled for other important confounders such as body mass index, reproductive factors received an additional star.

‡ One star was assigned if there was no significant difference in the response rate between control subjects and cases by using the chi-square test (*P* > 0.05).

### Dose-response analysis of saturated FA intake

**Figure 2:**
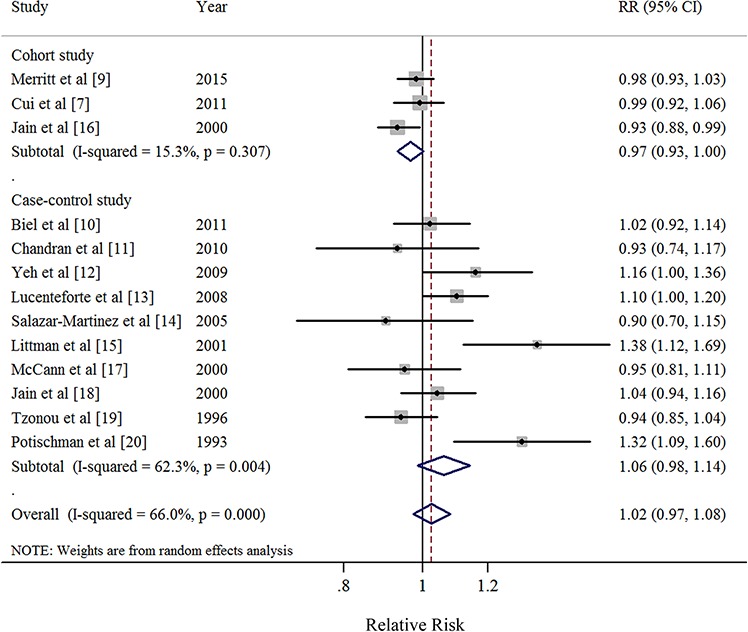
Forest plots (random effect model) of saturated fatty acid intake (per 10 g/day) and endometrial cancer risk by study design Squares indicate study-specific risk estimates (size of the square reflects the study-specific statistical weight); horizontal lines indicate 95% CIs; diamond indicates the summary relative risk with its 95% CI. RR: relative risk.

**Figure 3:**
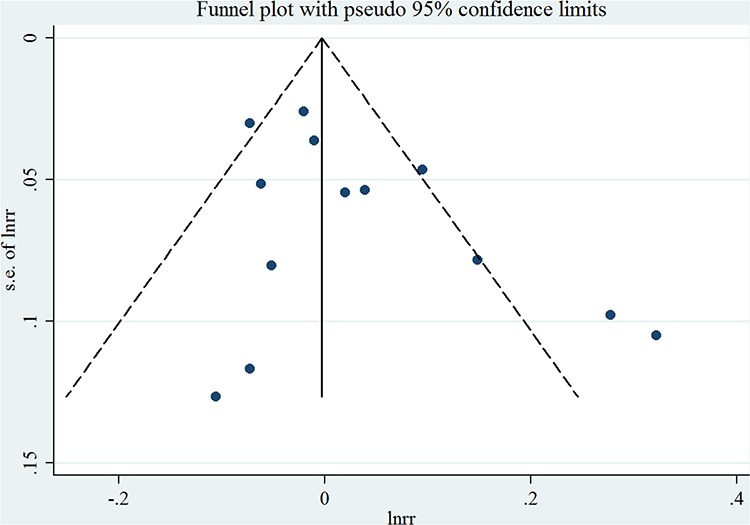
Funnel plot corresponding to the random-effects meta-analysis of the relationship between saturated fatty acid intake (per 10 g/day) and endometrial cancer risk

Thirteen studies [7, 9–20] were included in the dose-response meta-analysis of saturated FA intake and EC risk (Table 4). The summary RR for a 10g/day increase was 1.02 (95%CI = 0.97–1.08), with high heterogeneity (*I*^2^ = 66.0%, *P* for heterogeneity < 0.01) (Figure 2). No evidence of a potential nonlinear aforementioned association was observed (*P* for nonlinearity = 0.18). There was no indication of publication bias by visual inspection of the funnel plot (Figure 3) as well as by Egger's test (*P* for bias = 0.18) and Begg's test (*P* for bias = 0.30).

**Table 4:** Summary risk estimates of the association between dietary Saturated, monounsaturated, and polyunsaturated fatty acid intake and endometrial cancer risk, dose-response analysis (per 10 g/day increment)

	Saturated fatty acid					Monounsaturated fatty acid					Polyunsaturated fatty acid				
	No. of study	Summary RR (95%CI)	*I*^2^ value (%)	*P*_h_*	*P*_h_**	No. of study	Summary RR (95% CI)	*I*^2^ value (%)	*P*_h_*	*P*_h_**	No. of study	Summary RR (95% CI)	*I*^2^ value (%)	*P*_h_*	*P*_h_**
**Overall**	13	1.02 (0.97–1.08)	66.0	< 0.01		9	0.98 (0.96–1.001)	0	0.68		8	1.00 (0.95–1.06)	0	0.46	
**Study design**					0.17					0.87					0.62
Cohort study	3	0.97 (0.93–1.00)	15.3	0.31		3	0.97 (0.94–1.00)	0	0.87		2	1.00 (0.86–1.16)	54.0	0.14	
Case-control study	10	1.06 (0.98–1.14)	62.3	< 0.01		6	0.98 (0.95–1.01)	0	0.47		7	1.02 (0.94–1.09)	0	0.51	
Quality scores					0.81					0.49					0.80
High (≥9)	8	1.02 (0.96–1.07)	66.6	< 0.01		7	0.98 (0.96–1.01)	0	0.92		5	1.00 (0.94–1.07)	0	0.55	
Low (<9)	5	1.04 (0.90–1.20)	70.9	< 0.01		3	1.00 (0.89–1.11)	41.8	0.18		3	1.05 (0.82–1.36)	44.2	0.17	
**Geographic location**					0.66					0.36					0.28
North America	10	1.04 (0.97–1.12)	69.1	0.05		6	0.99 (0.96–1.03)	0	0.68		5	1.05 (0.94–1.16)	12.7	0.33	
Europe	3	1.00 (0.93–1.09)	67.8	< 0.01		3	0.97 (0.94–1.00)	0	0.45		3	0.98 (0.90–1.05)	0	0.69	
**Validated FFQ**					0.28					0.20					0.93
Yes	9	1.00 (0.96–1.05)	48.9	0.05		8	0.99 (0.96–1.01)	0	0.81		7	1.00 (0.94–1.08)	10.6	0.35	
No	4	1.12 (0.90–1.38)	83.3	< 0.01		1	0.95 (0.91–1.00)	N/A	N/A		1	1.01 (0.88–1.17)	N/A	N/A	
**Number of cases**					0.22					0.17					0.97
≥ 450	7	1.05 (0.99–1.12)	64.2	0.01		6	0.99 (0.96–1.02)	0	0.66		5	1.00 (0.94–1.07)	0	0.61	
< 450	6	0.98 (0.89–1.07)	58.5	0.03		3	0.95 (0.91–0.99)	0	0.95		3	1.03 (0.78–1.36)	50.2	0.13	
**Energy-adjusted model**					0.99					0.35					0.64
Yes	7	1.02 (0.96–1.10)	71.8	< 0.01		6	0.99 (0.95–1.01)	0	0.95		4	1.00 (0.90–1.11)	42.4	0.16	
No	6	1.03 (0.94–1.13)	64.0	0.02		3	0.98 (0.89–1.07)	43.5	0.17		4	1.03 (0.93–1.13)	0	0.74	
**Adjustment for potential confounders**															
**Total energy intake**					0.41					0.20					0.93
Yes	12	1.03 (0.98–1.09)	67.5	< 0.01		8	0.99 (0.96–1.01)	0	0.81		7	1.00 (0.94–1.08)	10.6	0.35	
No	1	0.94 (0.85–1.04)	N/A	N/A		1	0.95 (0.91–1.00)	N/A	N/A		1	1.01 (0.88–1.17)	N/A	N/A	
**Body mass index**					0.41					0.20					0.93
Yes	12	1.03 (0.98–1.09)	67.5	< 0.01		8	0.99 (0.96–1.01)	0	0.81		7	1.00 (0.94–1.08)	10.6	0.35	
No	1	0.94 (0.85–1.04)	N/A	N/A		1	0.95 (0.91–1.00)	N/A	N/A		1	1.01 (0.88–1.17)	N/A	N/A	
**Cigarette smoking**					0.62					0.48					0.83
Yes	10	1.03 (0.97–1.10)	68.8	< 0.01		6	0.99 (0.96–1.01)	0	0.62		5	1.00 (0.93–1.07)	0	0.55	
No	3	1.00 (0.88–1.13)	67.3	0.05		3	0.97 (0.93–1.01)	0	0.45		3	1.03 (0.87–1.21)	44.5	0.17	
**Parity**					0.57					0.20					0.93
Yes	11	1.02 (0.97–1.07)	59.0	0.01		8	0.99 (0.96–1.01)	0	0.81		7	1.00 (0.94–1.08)	10.6	0.35	
No	2	1.13 (0.77–1.64)	90.7	< 0.01		1	0.95 (0.91–1.00)	N/A	N/A		1	1.01 (0.88–1.17)	N/A	N/A	
**Oral contraceptive use**					0.71					0.43					0.80
Yes	10	1.03 (0.97–1.09)	69.6	< 0.01		6	0.98 (0.96–1.01)	0	0.91		5	1.00 (0.94–1.07)	0	0.55	
No	3	1.00 (0.86–1.17)	64.7	0.06		3	1.00 (0.89–1.11)	41.8	0.18		4	1.05 (0.82–1.36)	44.2	0.17	
**HRT use**					0.25					0.21					0.75
Yes	11	1.04 (0.98–1.10)	69.8	< 0.01		7	0.99 (0.96–1.01)	0	0.72		6	1.00 (0.94–1.07)	0	0.69	
No	2	0.93 (0.85–1.03)	0	0.75		2	0.95 (0.91–1.00)	0	0.83		2	1.53 (0.52–4.46)	71.7	**0.06**	

CI, confidence interval; FFQ, food frequency questionnaire; N/A, not available; RR, relative risk.

* *P*-value for heterogeneity within each subgroup.

** *P*-value for heterogeneity between subgroups with meta-regression analysis.

When stratified by study design, we found borderline significant result in cohort study (RR = 0.97; 95% CI = 0.93–1.00), with low heterogeneity (*I*^2^ = 15.3%, *P* for heterogeneity = 0.31). However, the non-significant results with high heterogeneity were observed in the majority of other subgroup analyses (Table 4). Furthermore, the results of meta-regression analyses did not show statistical significance. In a sensitivity analysis of saturated FA intake and EC risk, we sequentially removed one study at a time and re-analyzed the data. The 13 study-specific RRs ranged from a low of 1.01 (95% CI = 0.96–1.06, *I*^2^ = 57%, *P* for heterogeneity = 0.01) after omitting the study by Littman et al [15] to a high of 1.04 (95% CI = 0.98–1.10, *I*^2^ = 61.4%, *P* for heterogeneity < 0.01) after omitting the study by Jain et al [16].

### Dose-response analysis of monounsaturated FA intake

**Figure 4:**
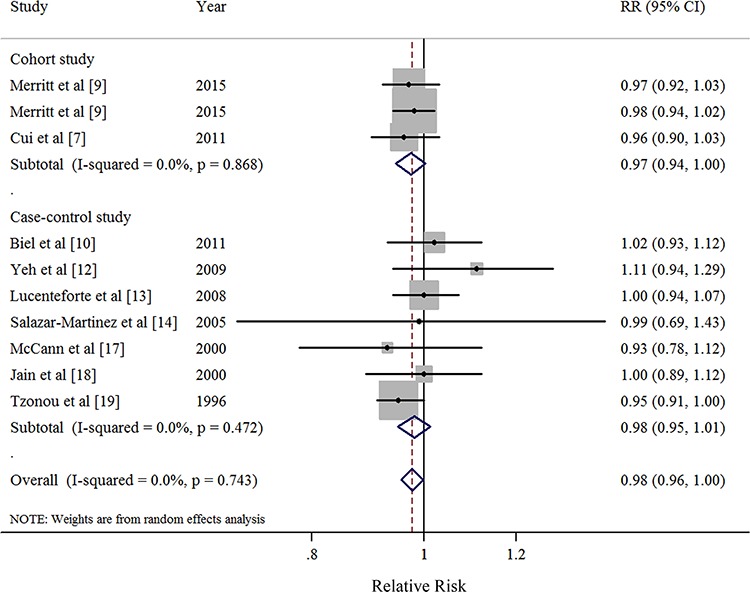
Forest plots (random effect model) of monounsaturated fatty acid intake (per 10 g/day) and endometrial cancer risk by study design Squares indicate study-specific risk estimates (size of the square reflects the study-specific statistical weight); horizontal lines indicate 95% CIs; diamond indicates the summary relative risk with its 95% CI. RR: relative risk.

**Figure 5:**
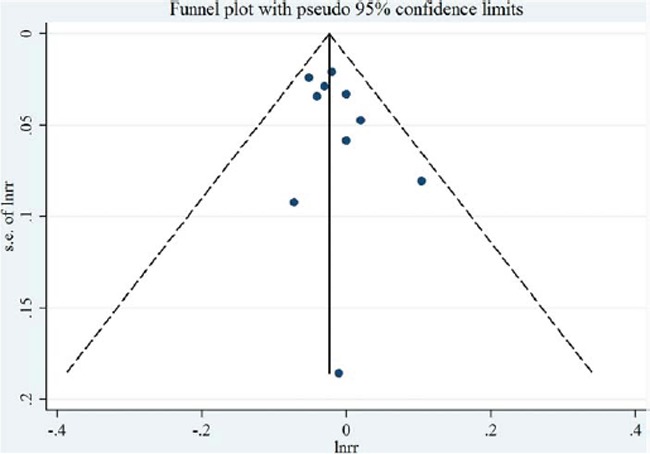
Funnel plot corresponding to the random-effects meta-analysis of the relationship between monounsaturated fatty acid intake (per 10 g/day) and endometrial cancer risk

Nine studies [9, 10, 12–14, 17–19] were included in the dose-response meta-analysis of monounsaturated FA intake and EC risk (Table 4). The summary RR for a 10g/day increase was 0.98 (95%CI = 0.96–1.001), without heterogeneity (*I*^2^ = 0%, *P* for heterogeneity = 0.68) (Figure 4). No evidence of a potential nonlinear aforementioned association was observed (*P* for nonlinearity = 0.87). There was no indication of publication bias by visual inspection of the funnel plot (Figure 5) as well as by Egger's test (*P* for bias = 0.25) and Begg's test (*P* for bias = 0.59).

The direction of the associations was consistent in the subgroup analyses stratified by study characteristics and adjustment for potential confounders. Furthermore, the majority of results showed borderline significance without or with little heterogeneity in the subgroup analyses except for these studies without using energy-adjusted model or without adjustment for oral contraceptive use (Table 4). The results of meta-regression analyses did not show statistical significance. In a sensitivity analysis of monounsaturated FA intake and EC risk, we sequentially removed one study at a time and re-analyzed the data. The 9 study-specific RRs ranged from a low of 0.98 (95% CI = 0.95–1.00, *I*^2^ = 0%, *P* for heterogeneity = 0.64) after omitting the study by Lucenteforte et al [13] to a high of 0.99 (95% CI = 0.96–1.01, *I*^2^ = 0%, *P* for heterogeneity = 0.81) after omitting the study by Tzonou et al [19].

### Dose-response analysis of polyunsaturated FA intake

**Figure 6:**
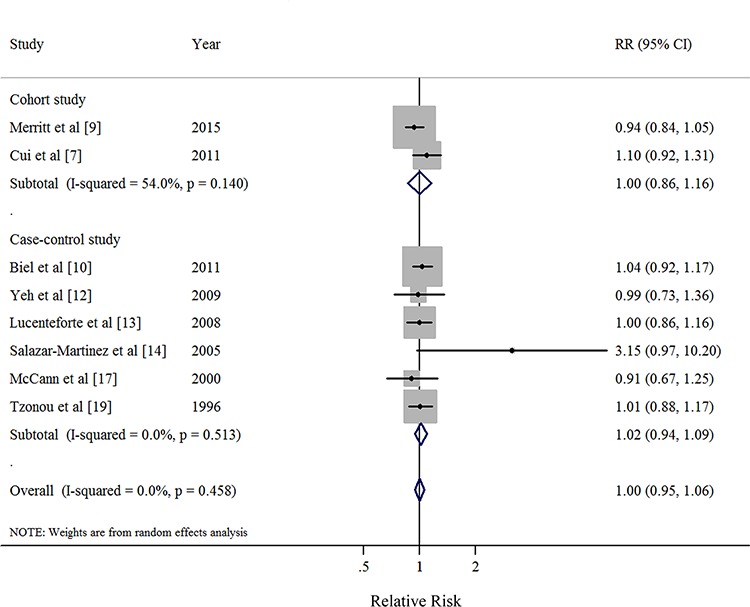
Forest plots (random effect model) of polyunsaturated fatty acid intake (per 10 g/day) and endometrial cancer risk by study design Squares indicate study-specific risk estimates (size of the square reflects the study-specific statistical weight); horizontal lines indicate 95% CIs; diamond indicates the summary relative risk with its 95% CI. RR: relative risk.

**Figure 7:**
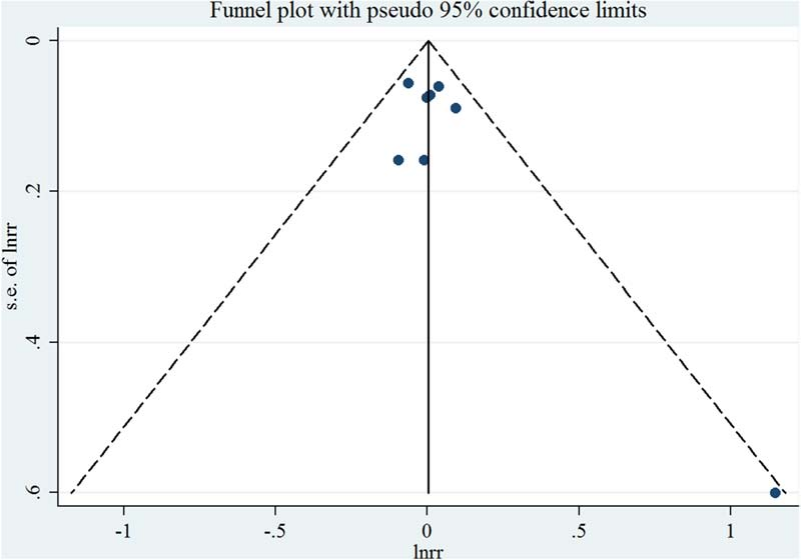
Funnel plot corresponding to the random-effects meta-analysis of the relationship between polyunsaturated fatty acid intake (per 10 g/day) and endometrial cancer risk

Eight studies [7, 9, 10, 12–14, 17, 19] were included in the dose-response meta-analysis of polyunsaturated FA intake and EC risk (Table 4). The summary RR for a 10g/day increase was 1.00 (95% CI = 0.95–1.06), without heterogeneity (*I*^2^ = 0%, *P* for heterogeneity = 0.46) (Figure 6). No evidence of a potential nonlinear aforementioned association was observed (*P* for nonlinearity = 0.14). There was no indication of publication bias by visual inspection of the funnel plot (Figure 7) as well as by Egger's test (*P* for bias = 0.17) and Begg's test (*P* for bias = 0.71).

When stratified by study characteristics and adjustment for potential confounders, the non-significant results were observed in all the subgroups (Table 4). The results of meta-regression analyses did not show statistical significance. In a sensitivity analysis of polyunsaturated FA intake and EC risk, we sequentially removed one study at a time and re-analyzed the data. The 8 study-specific RRs ranged from a low of 0.99 (95% CI = 0.93–1.06, *I*^2^ = 0%, *P* for heterogeneity = 0.47) after omitting the study by Cui et al [7] to a high of 1.03 (95% CI = 0.96–1.01, *I*^2^ = 0%, *P* for heterogeneity = 0.56) after omitting the study by Merritt et al [9].

## DISCUSSION

In this dose-response meta-analysis of 14 epidemiological studies, we found no statistically significant association between intake of saturated FA and EC risk. However, to the best of our knowledge, this is the first meta-analysis to report the relationship between monounsaturated and polyunsaturated FA intake and EC risk.

The present study is inconsistent with a previous meta-analysis which suggested that dietary saturated FA (summarized RR = 1.49; 95% CI = 1.11–2.01, *I*^2^ = 52.7%) intake was associated with an increased risk of EC [21]. However, this finding was based on the highest comparing with the lowest category of intake, which was hard to interpret because the definitions of the categories differed among these studies. For example, Potischman et al [20] reported the highest category (the fourth quartile) of saturated FA intake was over 25g/day in a population-based case-control study with 399 cases and 296 controls. However, the amount of saturated FA intake for the fourth quartile was over 37g/day with same population-based case-control study design from the same country [17].

Our meta-analysis has several strengths. To the best of our knowledge, this is the first dose-response meta-analysis systematically and quantitatively evaluates the association between dietary saturated, monounsaturated, and polyunsaturated FA intake and risk of EC. Moreover, compared with the previous meta-analysis [21], with six additional studies (two cohorts and four case-controls studies), our meta-analysis included a total of 7741 EC cases and 583,892 non-cases which provided sufficient statistical power to detect this putative association. Notably, we also carried out numerous subgroup and sensitivity analyses in this dose-response meta-analysis.

Despite the clear strengths of this study, some limitations of our study should be acknowledged. First, a meta-analysis is not able to solve problems with confounding factors that could be inherent in the included studies. Intake of diets high in FA may be associated with overweight and obesity, cigarette smoking and alcohol drinking, physical activity, and intake of total energy and other nutrients, which possibly could confound the aforementioned associations. Although the results for dietary FA intake persisted in studies that adjusted for these potential confounders, less of studies adjusted for alcohol drinking (*n* = 1) [11] and physical activity (*n* = 3) [11, 13, 14]. Therefore, we did not show these results which were difficult to interpret. Furthermore, since EC was a hormone-related cancer, we carried out the subgroup analyses stratified by adjustment for hormone-dependent risk factors (e.g., parity, oral contraceptive use, and hormone replacement therapy). The majority of included studies adjusted for these aforementioned confounders in their primary analyses. Additionally, the results of meta-regression analyses found no evidence that these findings differed significantly between studies adjusted for these confounders or not. Second, measurement errors in the assessment of dietary intake are known to bias effect estimates [22]; although all included studies evaluated the dietary intake through food frequency questionnaire (FFQ), none of them made any corrections for measurement errors. Any measurement errors would most likely result in bias toward the null, which underestimate the relationship between dietary FA intake and EC risk. Moreover, because the estimation of dietary FA intake was based on the FFQ which might vary among these include studies. For example, the FFQ of the EPIC contained up to 260 food items [9]. In contrast, Yeh et al [12] measure the dietary intake on the basis of 44-item FFQ in a hospital-based case-control study. Third, although the meta-regression analyses did not show statistical significance, the point estimates were slightly different between cohort and case-control studies (0.98 *vs*. 1.06), which could be partly attributed to the methodological differences in study designs. Compared with case-control studies, prospective studies are less susceptible to bias (e.g. recall bias, selection bias) due to their nature. Additionally, on the basis of the updated NOS, less case-control studies fulfilled these criteria than cohort studies (Table 2 and Table 3). Therefore, further prospective studies are warranted to confirm our findings. Last, since women who have had a hysterectomy are at virtually no risk of developing EC, none of the studies updated hysterectomy status of their population during follow-up which might bias results toward the null [21].

In summary, our dose-response meta-analysis provides limited evidence that dietary intake of saturated, monounsaturated, and polyunsaturated FA was associated with the risk of EC. Since few prospective studies were included, the findings of this study are warranted to be confirmed. Additionally, further studies are needed to provide more detail results, including those for other type of dietary FA and stratify the results by the histology of EC after better adjustment for the potential confounding.

## MATERIALS AND METHODS

### Search strategy

Two independent investigators (Q-JW and T-TG) systematically searched PubMed, EMBASE, and Web of Science from each database's inception to the end of June, 2015 to identify relevant epidemiological studies. The following search keywords were used: (diet OR dietary OR fat OR fatty OR fatty acid) AND (endometrium OR endometrial) AND (cancer OR tumor OR carcinoma OR neoplasm). This search strategy was similar to previous studies [23–25]. We followed the preferred reporting items for systematic reviews and meta-analyses (PRISMA) guidelines to plan, conduct and report this meta-analysis [26].

### Study selection and exclusion

To be included in this analysis, a study must have (i) an observational study design; (ii) evaluated the association between dietary FA (saturated, monounsaturated, and polyunsaturated FA) intake and EC risk; and (iii) presented RR, odds ratio (OR), or hazard ratio (HR) estimates with 95% CIs or necessary data for calculation [23]. If several publications involved overlapped individuals, we included the study with the most patients.

The studies were excluded by the following exclusion criteria: (i) were randomized controlled trials, reviews without original data, ecological studies, editorials, and case reports; (ii) reported the risk estimates that could not be summarized (such as reported the risk estimates without 95% CIs); and (iii) reported the outcome as EC mortality or recurrence [23].

### Data extraction and quality assessment

Data were extracted by two investigators (Q-JW and T-TG) using a data extraction form and entered into a database. All differences were resolved by discussion with third investigator (Y-ZW). For each included study, we extracted the following information: last name of the first author, publication year, geographic location, number of cases/controls (size of cohort), exposure assessment and categories, and study-specific adjusted estimates with their 95% CIs for the highest compared with the lowest category of intake (including adjusted confounders information if applicable). If there were multiple estimates for the association, we used the estimate adjusted for the most appropriate confounding variables, like previous studies [23, 27–29].

An update Newcastle-Ottawa Scale (NOS) [23, 29–31] uses four quality parameters including selection, comparability, exposure/outcome, and energy-adjusted model was used to assess the methodological quality of all included studies. The full score was 10 and the high-quality study was defined as a study with quality scores ≥ 9.

### Statistical analysis

As the absolute risk of EC is low and therefore we interpreted all risk estimates as relative risk (RR) for simplicity [23]. For study [9] reported aforementioned associations on the basis of the EPIC as well as the NHS/NHSII but in one article, we treated it as two included studies. For study [19] did not provide the adjusted risk estimate, we used the exposure distribution of cases and controls to calculate the crude risk estimate.

To examine the associations between the dietary FA intake and EC risk, the summary RR with 95% CIs were estimated by summarizing the risk estimates of each study using the random effect models, which considered both within- and between-study variation [32]. We summarized the study-specific RR for each 10 g/day increment in dietary FA intake. The study-specific trend from the correlated log RR across the categories of dietary FA intake was computed by using the generalized least-squares trend estimation method developed by Greenland and Longnecker [33] and Orsini et al [34]. For studies reported the risk estimates as per standard deviation (SD) increment of total FA intake, we used previously described methods [35, 36] to recalculate risk estimates into per 10g/day increment which was suggested by the continuous update project of WCRF/AICR [8]. Furthermore, a potential nonlinear dose-response relationship between the dietary FA intake and the EC risk was modeled by using restricted cubic splines with three knots at fixed percentiles (10, 50 and 90%) of the distribution of exposure. We calculated the overall *P*-value by testing that these two regression coefficients were simultaneously equal to zero. We calculated a *P*-value for nonlinearity by testing that the coefficient of the second spline was equal to zero. The details of this method has been published elsewhere [37, 38].

For conducting the dose-response meta-analysis, the following information were needed: (i) the distribution of cases and non-cases and the risk estimates with the variance estimates for at least three quantitative exposure categories; (2) the median or mean level of these exposures in each category (if reported by ranges, mean level was calculated by averaging the lower and upper bound; if the lowest category was open ended, the lowest boundary was considered to be zero; if the highest category was open ended, the open-ended interval length was assumed to be the same as the adjacent interval). Given this, thirteen, nine, and eight studies met the criteria and were included in the dose-response analysis of saturated, monounsaturated, and polyunsaturated FA intake and EC risk, respectively.

To investigate the possible sources of heterogeneity of main results, we carried out stratified analyses by the following study features: study design (cohort *versus* case-control studies), quality scores (high *versus* low), geographic location (North America *versus* Europe), validated food frequency questionnaire (yes *versus* no), mean number of EC cases (≥450 *versus* < 450), energy-adjusted model (yes *versus* no), and adjustment for potential confounders including total energy intake, body mass index, cigarette smoking, parity, oral contraceptive use, menopausal status, and hormone replacement therapy use. Heterogeneity between subgroups was evaluated by meta-regression [23, 29–31].

Small study bias, such as publication bias can reflect genuine heterogeneity, chance, or other reasons for differences between small and large studies which was evaluated with Egger's linear regression asymmetry test [39] and Begg's rank-correlation test [40]. A *P*-value of 0.05 was used to determine whether significant publication bias existed. Furthermore, sensitivity analyses were conducted by deleting each study in turn to reflect the influence of individual data on the overall estimate. All statistical analyses were performed with Stata (version 12; StataCorp, College Station, TX).
